# Selected Medicinal Plants Used in the Treatment and Management of Tuberculosis and Related Symptoms in South Africa

**DOI:** 10.3390/ph18040513

**Published:** 2025-03-31

**Authors:** Makosha P. Mamabolo, Babalwa Tembeni, Xavier Siwe Noundou, Nontobeko P. Mncwangi

**Affiliations:** 1 Department of Pharmaceutical Sciences, School of Pharmacy, Sefako Makgatho Health Sciences University, MEDUNSA, P.O. Box 218, Ga-Rankuwa 0204, South Africa; 2 African Genome Center, Mohammed VI Polytechnic University, Lot 660, Hay Moulay Rachid, Ben Guerir 43150, Morocco; btembeni2@gmail.com; 3 Department of Pharmacy Practice, School of Pharmacy, Sefako Makgatho Health Sciences University, MEDUNSA, P.O. Box 218, Ga-Rankuwa 0204, South Africa; nontobeko.mncwangi@smu.ac.za

**Keywords:** antimycobacterium activity, tuberculosis, South Africa, medicinal plants

## Abstract

**Background/Objectives**: Medicinal plants are used around the globe to treat and/or manage various medical conditions, including respiratory diseases such as tuberculosis, which affect the lower respiratory tract, with its related symptoms being treated and/or managed using medicinal plants. This review collates the available literature pertaining to the medicinal uses and phytochemistry of *Carpobrotus edulis*, *Drosera capensis*, *Pelargonium reniforme*, and *Tulbaghia violacea* used for the treatment and management of tuberculosis in South Africa. The abovementioned plants were selected based on their long history of use, anecdotal evidence, and the scientific data available. **Methods**: Data to compile this review article were sourced and analyzed from Google Scholar, Pubmed, ScienceDirect, and textbooks published from 2000 to 2022. The search terms included the plant and genus names of each species, tuberculosis, and *Mycobacterium tuberculosis*. **Results**: The data obtained indicate that the plants do not only have an effect on *Mycobacterium tuberculosis*, but also on other conditions, including cough, colds, eczema, infections, and asthma, which are differential diagnoses in suspected tuberculosis cases. The literature indicates that extracts from the four plants under review have antimicrobial activity, with MICs ranging between 0.20 and 50.00 mg/mL. The major classes of phytochemicals identified from the four medicinal plants included flavonoids, naphthoquinone, terpenoids, and sulfur-containing compounds. **Conclusions**: The literature review on the plants reveals that they are also used to treat other lower-respiratory ailments, including cough and fever, which may be signs and symptoms of TB. The literature review reveals that medicinal plants contain valuable phytochemicals which may be strong drug leads to combat the tuberculosis epidemic.

## 1. Introduction

Tuberculosis (TB) is an infectious disease caused by *Mycobacterium tuberculosis*; it can cause a silent, latent, or progressive active infection [1]. In a 2022 report, the World Health Organization estimated that a quarter of the world’s population (approximately 2 billion people) are infected with tuberculosis, with about 2–3 million people dying from TB each year [1,2]. However, some people will not develop the TB disease, and thus will not transmit it, and some will clear the infection [3]. In 2020 alone, it was reported that a total of 1.5 million people died from TB [3]. In 2021, 10.6 million people fell ill with TB and approximately 1.6 million people died [3].

The United States Food and Drug Administration (US-FDA) has ten approved drugs for TB, and, of those, four drugs form a part of the first-line treatment of TB, namely isoniazid, rifampicin, ethambutol, and pyrazinamide [4,5]. The latest drug approved for use by the FDA was approved in 2019, a drug called pretomanid, which is also the third new drug approved by the administration in over 40 years [6]. The WHO recommends that new TB patients presumed to have drug-susceptible TB should receive six months of drug treatment with the first two months, being intensive treatment followed by a four-month continuation treatment, as summarized in Table 1 [7]. The initial phase is the bactericidal phase, which ensures that the *Mycobacterium* with a high rate of replication are eradicated, thus reducing symptoms and resolving clinical signs [8]. The continuation phase is the sterilizing phase when semi-dormant bacteria are eliminated, thus lowering the probability of the emergence of drug-resistant *Mycobacterium* [8]. Multi-drug-resistant tuberculosis (MDR-TB), however, can be treated up to a period of two years [9]. The intensive treatment involves taking isoniazid, rifampicin, pyrazinamide, and ethambutol. The continuation treatment involves taking isoniazid and rifampicin. It is vital that the TB drugs are taken together at a given time due to the possibility of quickly developing drug resistance [7]. The treatment should be taken every day for six months, and the course should be completed. The treatment is rendered ineffective if it is interrupted or stopped early due to the ability of *M. tuberculosis* to develop resistance rapidly against TB drugs [7]. The drug regimen, as well as its frequency, is dependent on whether the patient is an adult or child, whether the patient is infected with Human Immunodeficiency Virus (HIV) or not, and whether the patient has cavitation on an initial chest x-ray and positive cultures at completion of two months of therapy [7]; these factors will determine which patient receives which regimen. Table 1 and Table 2 adopted from the Center for Disease Control and Prevention (CDC) indicate the recommended initial and continuation phase drug regimen for drug susceptible pulmonary TB.

**Figure 1:**
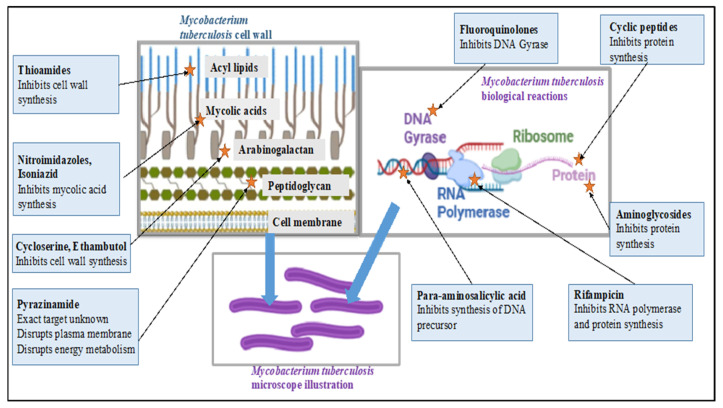
Summary of the mechanisms of action for current TB drugs. Figure was illustrated using BioRender^®^ and inspired by [10].

An illustration of the mechanism of action for current TB drugs is depicted in Figure 1.

Ethnobotanically, traditional practitioners ‘diagnose’ tuberculosis by visual examination of the signs they observe visible of the patients and symptoms reported by patients such as cough, weight loss, and night sweats [11]. The aim of the review is to highlight the traditional uses of the selected four plants, *C. edulis*, *D. capensis*, *P. reniforme*, and *T. violacea*, based on the reported studies conducted on the plant extracts in the literature, specifically antimycobacterium studies including isolated or present phytochemicals.

## 2. Literature Search Strategy

The applications of four medicinal plants were sourced from different sources, including Google Scholar, Pubmed, Pubchem, and Sciencedirect, as well as textbooks dated between 2000 and 2022, with one report from 1992 that specified isolated compounds. This period was selected in order to obtain sufficient and relevant data, as well as to highlight the improvements in research over the years. A desktop review was conducted using the following approach: Firstly, scientific publications were screened. In appraising the available body of knowledge, the keywords tuberculosis, phytochemistry, *Carpobrotus edulis* (L.) Bolus., *Drosera capensis*, *Pelargonium reniforme*, *Tulbaghia violacea*, and toxicology had to be contained within the publications reviewed. The software ChemDraw Professional^®^ v15.0 was used to illustrate the chemical structures of the phytochemicals present in the different plant extracts. The Mendeley referencing tool was used to cite the in-text references, as well as to compile the bibliography. Section 2.1, Section 2.2, Section 2.3 and Section 2.4 provide the botanical descriptions, medicinal uses, pharmacological effects, phytochemistry (including isolated compounds), and studies performed on the plant extracts (including in vivo, in vitro, and clinical data) where available for the four selected plants.

### 2.1. Botanical Description of Carpobrotus edulis (L.) Bolus

**Figure 2:**
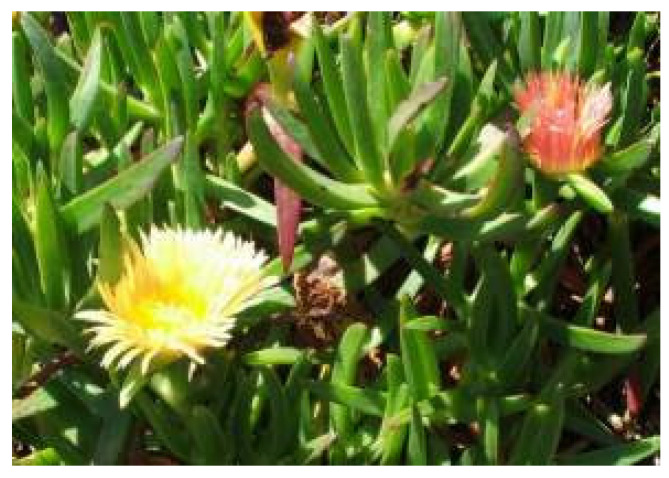
*Carpobrotus edulis* (L.) Bolus [16].

*Carpobrotus edulis* (L.) Bolus is an edible succulent that belongs to the family Aizoaceae or Masembryantgemaceae naturally distributed in the Western, Eastern, and Northern Cape [12,13] The family comprises 143 genera and about 2300 species across the tropical and sub-tropical regions [14]. The plant *C. edubilis* is commonly known as sour fig, Cape fig, and Hottentots fig in English, ikhambi-lamabulawo and umgongozi in IsiZulu, igcukuma in Xhosa, ghaukum in Khoi, and ghoenavy, hottentotsvy, kaapsevy, perdevy, rankvy, suurvy, and vyerank in Afrikaans [12,13,15]. It is a perennial mat-like creeper succulent with smoothly upright and triangular-shaped fleshy leaves, as visualized in Figure 2. It has large and fleshy yellow flowers that develop into aromatic fleshy fruits with a jelly like sour-sweet fruit pulp and a multitude of small brown seeds. The ripe fruits are famously used for jams and curry dishes and are sold in the Cape’s street markets [13].

#### 2.1.1. Ethnobotanical Uses of *Carpobrotus edulis*

The leaf juice is traditionally used to treat tuberculosis and is gargled to treat mouth and throat infections [13,17]. It is taken orally to treat digestive ailments and dysentery. The plant juice is used as a diuretic and a styptic, applied on the source to treat eczema, wounds, and burns. The leaf pulp is reported to treat toothache, earache, and oral and vaginal thrush [13]. The plant is also used to treat tuberculosis, diabetes, high blood pressure, toothache, headaches, oral and vaginal thrush, intestinal worms, constipation, sores, and infections of HIV/AIDS [15]. Other reported uses of the plant include sinusitis, diarrhea, spider and tick bites, infantile eczema, and fungal and bacterial infections [18]. The traditional uses are summarized in Table 3.

#### 2.1.2. Biological Effects of *Carpobrotus edulis*

Antimicrobial, antioxidant, and antifungal effects are reported for *C. edulis*. Aqueous leaf extracts reportedly show antibacterial activity against *Staphylococcus aureus* and *Pseudomonas aeruginosa*, with minimum inhibitory concentration (MIC) values between 4.00 and 6.50 mg/mL, as well as antioxidant activity when using the DPPH method [13,17]. It is reported that antibacterial MIC values for extracts are considered significant when equal or less than 0.1 mg/mL, moderate at greater than 0.1 mg/mml, but less than 0.625 mg/mL, and weak if greater than 0.625 mg/mL [19], and, thus, the reported MICs indicate weak efficacy or lack of efficacy thereof. Methanolic extracts below a toxic level have shown activity against multidrug-resistant *Mycobacterium tuberculosis*, as well as methicillin-resistant *S. aureus* [20]. The extracts inhibit the growth of multidrug-resistant *M. tuberculosis* within three days of culture and methicillin-resistant *S. aureus* within six hours of culture [20]. In another study, the antimicrobial effect of methanolic leaf extract against *Moraxella catarrhalis* was reported to have a concentration of 50.00 mg/mL using an agar plate diffusion assay [21]. Cytotoxicity studies were performed on Raw 264.7, Vero Kidney, and HepG2 cell lines, with the results showing LC_50_ ranging between 89.98 ± 10.29 to 849.86 ± 7.13 µg/mL, thus indicating a safe profile for use in humans [22].

#### 2.1.3. Phytochemistry of *Carpobrotus edulis*

*Carpobrotus edulis* is reported to contain alkaloids, flavonoids, flavonols, phenolics, proanthocyanidins, saponins, and tannins [13,17]. The presence of tannins supports the use of the plant as an antiseptic and strong astringent. Other active components that are reportedly present in the plant are catechin, malic acid, citric acid, ferulic acid, hyperoside, rutin, and neohesperidin [13]. A report by Omoruyi, Bradley, and Afolayan, 2012, indicates the strong presence of phenolics, tannins, and proanthocyanidins, as well as a moderate presence of alkaloids and saponins [15]. The essential oils analyzed using gas chromatography–mass spectrometry (GC-MS) revealed a composition of monoterpenes, sesquiterpenes, diterpenes, and fatty acids [23]. Another report revealed isolated compounds from the methanolic extract using column chromatography and further purified using reverse-phase high-performance liquid chromatography, including β-amyrin (**1**) oleanolic acid (**2**), uvaol (**3**), monogalactosyldiacylglycerol (MGDG) (**7**), catechin (**6**), epicatechin (**4**), and procyanidin B5 (**13**), which were identified using NMR and a comparison of the spectral data with those published in the literature [24]. Table 4 summarizes the isolated and identified compounds; however, there are no reports for mechanism of action of compounds on the *M. tuberculosis* pathogen.

**Table 3:** The uses, biological effects, and phytochemistry of *Carpobrotus edulis*.

Plant Part	Uses	Extraction		Biological Effect	Phytochemistry		References
	Traditional	Method	Type of Extract		Analysis/Profile	Bioactive Components	
Leaf juice Leaf pulp	Mouth and throat infections, dysentery, digestive troubles, TB, diuretic and styptic, eczema, wounds and burns, toothache, earache, oral and vaginal thrush Wounds and infections	NR	NR	Antimicrobial activity	NR	Catechin (**6**), malic acid (**9**), citric acid (**8**), ferulic acid (**5**)	[13]
Leaves	TB, sore throat, lung infections	NR	NR	Antimicrobial activity	TLC	Tannins and flavonoids, Hyperoside, rutin, neohesperidin	[13,17]
		Centrifugation	Methanolic extract	Inhibits the growth of multidrug-resistant *M. tuberculosis* within three days of culture and methicillin-resistant *S. aureus* within six hours of culture below toxic levels	NR	NR	[20]
		Maceration with stirring followed by centrifuging	Methanolic extract	Antimicrobial activity against *M. catarrhalis* with concentration of 50 mg/mL	NR	NR	[21]
			Methanolic extract	Antiproliferative activity	Column chromatography	β-amyrin (**1**), oleanolic acid (**2**), uvaol (**3**), monogalactosyldiacylglycerol (**7**) (MGDG), catechin (**6**), epicatechin (**4**), and procyanidin B5 (**13**)	[25]

NR—Not reported at this time.

### 2.2. Botanical Description of Drosera capensis L.

**Figure 3:**
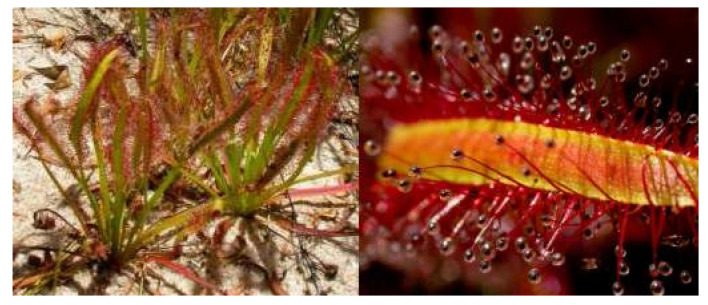
*Drosera capensis* [64].

*Drosera capensis* belongs to the family Droseraceae and is distributed in the Eastern and Western Cape, with 160 species within the *Drosera* genus [17,61]. *D. capensis* is a small upright perennial plant (Figure 3), commonly known as Cape sundew in English, and is native to the Cape of South Africa [62,63]. The cape sundew is a carnivorous plant that uses sticky tentacles to capture their prey [62].

#### 2.2.1. Ethnobotanical Uses of *Drosera capensis*

*Drosera capensis* is used as a traditional remedy for fever and tuberculosis [17]. It has also been used to treat warts, corns, sunburn, asthma, coughs, eye and ear infections, liver pain, morning sickness, stomach conditions, syphilis, toothache, and intestinal problems [64].

#### 2.2.2. Biological Effects of *Drosera capensis*

The antimicrobial activity of the leaf ethanol extract was investigated against *Mycobacterium smegmatis* as well as *Mycobacterium tuberculosis*, with inhibition indicated in *M. smegmatis* with MIC 3.12 mg/mL and no activity in *M. tuberculosis* [65]. *Drosera capensis* is reportedly not toxic; however, when taken in large quantities, it can result in the irritation of the digestive tract lining, thus causing stomach pains or gastritis [66]. However, there is no reports that states recommended dose. The cytotoxicity of *D. capensis* was investigated on Vero cells with IC_50_ of 141.40 µg/mL [65]. The traditional uses of the plants and their biological effects are summarized in Table 5.

#### 2.2.3. Phytochemistry of *Drosera capensis*

Flavonoids, including Quercetin, Myricetin, and Leucocyanidin, are reported as the bioactive components in the plant [17]. Compounds identified in *D. capensis* and the activity against *M. tuberculosis* are summarized in Table 6, including Plumbagin, which was identified in the methanolic extracts [67]. Other phytochemicals identified in *D. capensis* included 7-Methyljuglone (**45**), Mamegakinone (**46**), Neodiospyrin (**47**), Quercetin (**48**), Myricetin (**49**), Leucocyanidin (**50**), Leucopelargonidin (**51**), Leucodelphinidin (**52**), and Ellagic acid (**53**) [68]. The antimycobacterium mechanism of action was reported in two compounds—Plumbagin, a Naphthoquinone, and Quercetin, a flavonoid—both of which have anti-inflammatory properties [69,70]. TB infection in the lungs cause mild inflammation [71], thus confirming that the plant can help in the management of TB.

### 2.3. Botanical Decription of Pelargonium reniforme Curtis

**Figure 4:**
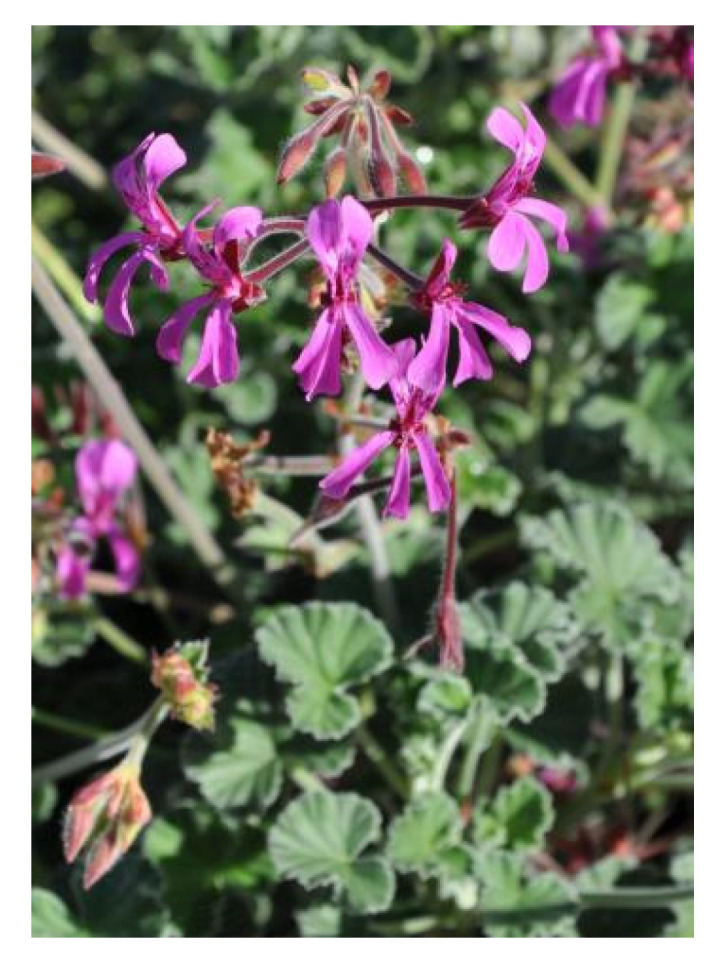
Pelargonium reniforme Curtis [81].

*Pelargonium reniforme* Curtis belongs to the Geraniaceae family, which consists of 5 genera and 830 species [81,82]. The plant is commonly found in the Eastern Cape, ranging from Knysna to Umtata. It is commonly known as kidney-leaved pelargonium in English, rooirabas in Afrikaans, and iyeza lesikhali and umsongelo in IsiXhosa [81]. It is a small upright perennial shrublet with tuberous roots that grow about 300–400 mm in height, but which have been known to reach 1 m. They have kidney- or heart-shaped leaves, with the mat-like hairs on the leaves being responsible for the plant’s velvety texture and gray-green color, as observed in Figure 4 [81].

#### 2.3.1. Ethnobotanical Uses of *Pelargonium reniforme* Curtis

The plant is used traditionally as a remedy for stomach ailments, bronchitis, and bloody stools [81,83]. The Xhosa and Zulu tribes in South Africa use the plant to treat cough, tuberculosis, dysentery, and diarrhea [17,84,85]. The plant is also used to manage menstrual complaints [86].

#### 2.3.2. Biological Effects of *Pelargonium reniforme*

The antimicrobial effects of the tuber against *M. tuberculosis* were investigated for the acetone, chloroform, and ethanol extracts by evaluating the minimum inhibitory concentration (MIC) of the plant extracts with an MIC value of 10.30 mg/mL [17], as summarized in Table 7. The antioxidant activity of the isolated bioactive compounds was investigated using the DPPH method, with IC_50_ ranging from 2.60 to 32.90 µM, with ascorbic acid standard IC_50_ of 40.0 µM [87]. The toxicology of the plant was investigated on the aqueous extract, which indicated no possibility of the toxicity of the hematological parameters in rats, and thus could be safe for use as a traditional medicine [86].

#### 2.3.3. Phytochemistry of *Pelargonium reniforme*

Phenolic compounds, including coumarins and scopoletin, are reported as bioactive compounds in *P. reniforme* [17]. Bioactive components isolated (Table 8) from *P. reniforme* included gallic acid (**54**), methyl gallate (**55**), glucogallin (**56**), corilagin (**57**), vitexin (**58**), isovitexin (**59**), orientin (**60**), isoorientin (**61**), vitexin 2″-gallate (**62**), quercetin (**63**), isoquercitrin (**64**), and rutin (**65**) [87]. Table 8 also summarizes the biological activity of the various compounds.

### 2.4. Botanical Description of Tulbaghia violacea Harv.

**Figure 5:**
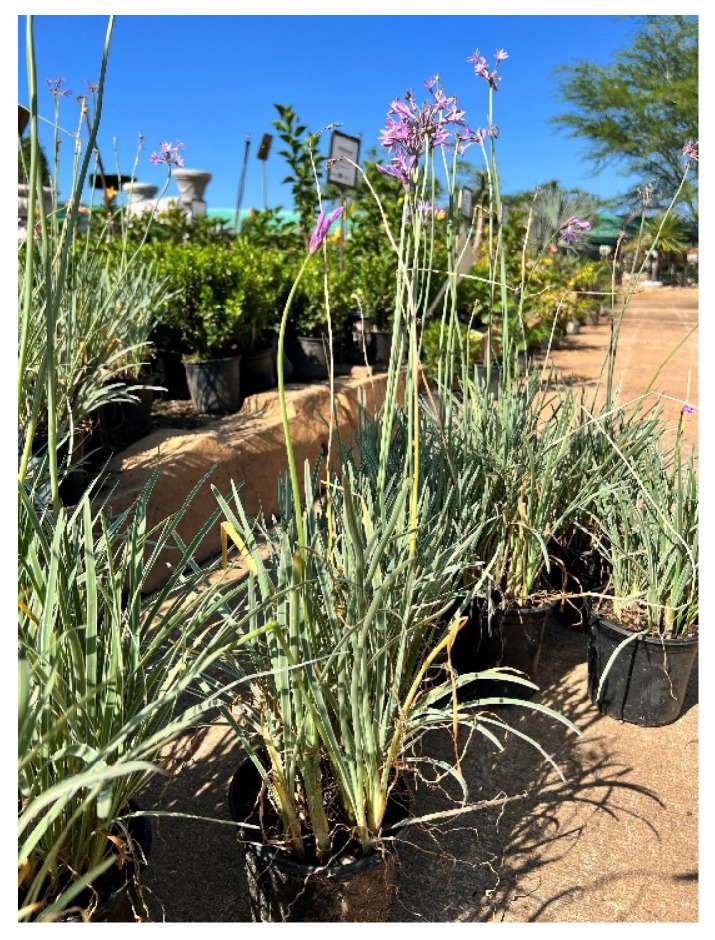
*Tulbaghia violacea* (picture captured with cellphone camera).

*Tulbaghia violacea* belongs to the Alliaceae family and is distributed in the Eastern Cape as well as southern Kwa-Zulu Natal [13]. *Tulbaghia* genus has 63 species, with 21–30 species mostly found in Southern Africa. The species in this genus are characterized by an onion or garlic odor that comes from the leaves when they are cut [100,101]. The plant *T. violacea* is commonly known as wild garlic in English, isihaqa in Zulu, and wilde knoffel in Afrikaans. It is a bulbous plant that has hairless narrow leaves growing from white fleshy bases. The plant has a strong garlic smell when bruised. It has purple flowers, as depicted in Figure 5, which occur as a group at the top of the plant’s stalk [13].

#### 2.4.1. Ethnobotanical Uses of *Tulbaghua violacea*

The bulbs and leaves of the plant are used as a traditional remedy for fever and colds, as well as asthma, lung ulcerations, sinusitis, and tuberculosis [13,102]. A decoction of the plant, prepared by boiling the bulbs in water, is used as an enema for stomach problems, and the leaves, which can be eaten as a vegetable, are used to treat esophagus cancer [13,17]. The leaves can also be used as a tick, flea, and mosquito repellent [103]. Furthermore, the plant is used by Zulu traditional healers to treat bronchitis and asthma [104].

#### 2.4.2. Biological Effects of *Tulbaghia violacea*

Antibacterial, antifungal, and antihypertensive effects were reported for *T. violaceae*. The dichloromethane bulb extract indicated antibacterial effects against *Klebsiella pneumonia* and *S. aureus*, with a minimum inhibitory concentration value of 0.195 mg/mL [13,17]. The bulb of the plant indicated an antifungal effect on *Candida albicans* [105]. The antibacterial activity of essential oils isolated from *T. violacea* exhibited activity against *Pseudomonas aeruginosa*, *Streptococcus faecalis*, *Acinetobacter calcoaceticus anitratus*, *Bacillus subtilis*, *Enterococcus faecalis*, *Staphylococcus aureus*, and *Streptococcus viridans* [104]. Cytotoxicity studies have been conducted on the plant, including its effect against Vero cells, which yielded results of 0.4909 ± 0.034 mg/mL, indicating a non-toxic profile against normal cells [106].

#### 2.4.3. Phytochemistry of *Tulbaghia violacea*

The plant reportedly contains sulfur compounds, allin, and *S*-(methylthiomethyl)-cysteine-4-oxide, which is the main compound in the intact plant and is broken down to marasmicin [13]. Sulfur-containing compounds were isolated and characterized from the plant [107]. Essentials oils extracted from *T. violacea* were subjected to GC-MS, revealing a number of volatile constituents including acetamide, 2-cyano (**69**), chlorodifluoro acetamide (**68**), σ-xylene, (E)-2-heptenoic acid (**70**), ρ-xylol, ρ-xylene thiodiglycol (**72**), 2,4-dithiapentane (**73**), chloromethylmethyl sulfide (**74**), acetamide (**71**), phthalic acid 2-ethylhexyl isobutyl ester (**77**), phthalic acid (**75**), phthalic acid heptyl2-methylallyl ester (**76**), nonadecane (**78**), heptacosane (**79**), and tetracosane (**80**) [104]. The biological activities of the plant are provided in Table 9. However, these are mainly on crude extracts. Table 10 indicates the compound names of the compounds identified in the plant; however, the phytochemical profile, biological activity, specific anti-TB assay data, and the proposed mechanism of action are unavailable, or are otherwise scarse, for this plant. The activity of specific compounds, especially against *M. tuberculosis*, requires further investigation.

## 3. Conclusions

The long-term use of medicinal plants for generations clearly indicates the importance of these naturally occurring agents in our society.

- From this review, it can be concluded that the four plants are not only used to treat or manage tuberculosis. They are also used to treat other lower-respiratory ailments, including cough and fever, which may be signs and symptoms of TB.
- From the four commonly used plants, the important phytochemicals were identified from different plants, including flavonoids, phenols, terpenes, naphthoquinones, and phenolics. The mentioned phytochemicals are generally abundant in nature and are also found in other plants.
- *Carpobrotus edulis* and *Tulbaghi violacea* are edible plants, and, because of this fact, it may be safe to conclude that the plant can be taken or formulated as supplements or nutraceuticals. Many more plants are taken as teas, such as Senna, Green tea, and Rooibos. The teas contain phytochemicals such as flavonoids. Therefore, the plants may most probably be taken as tea, if edible, to manage illnesses.
- The South African Health Products Regulatory Authority (SAHPRA) has a committee dedicated to evaluate complementary medicine. It will then be recommended to submit products for evaluation with the safety and efficacy profiles.
- There are reports that provide evidence that phytochemicals, including alkaloids, flavones, phenols, terpenoids, and some fatty acids, are effective against *Mycobacterium* strains [111]. Most of these phytochemicals were identified in the four plants, and thus there is scientific evidence that these plants and the isolated compounds from them could serve as potential drug candidates for new anti-TB drugs. However, there are no reports beyond the potential drug candidates. It is important to note that basic research has a great impact in assembling knowledge and there is, therefore, a need to report data in a systemic manner.
- Plumbagin (**44**) is one of the most effective isolated compounds and was identified in *D. capensis* as per the review. Plumbagin (**44**) is effective against MDR and XDR tuberculosis [112].
- From Table 10, it is clear that further research can be conducted on compounds identified in *T. violacea* to investigate their efficacy against TB, as well as their mode of action. In fact, Table 4, Table 6, Table 8 and Table 10 indicate that there is an opportunity to further investigate which specific compounds are responsible for the effects against *M. tuberculosis*. Further research into these plants may provide treatments for TB, as well as the management or treatment of the signs and symptoms of TB, including the clinical safety and efficacy aspects.
- Many plants are commercialized without any scientific evidence, which poses a danger to society. It is therefore important for SAHPRA, as well as other Medicine Regulatory Authorities (MRAs), around the globe to develop frameworks that guide the assessment of the safety, efficacy, and quality of traditional medicines, as well as to have a harmonized regulatory standard amongst the various MRAs.
- Generally, there is an assumption that the use of traditional medicine is safer than modern medicine. Therefore, there is a need to educate the public regarding the safe use of medicinal plants. Some plants are toxic and can be fatal when taken in large quantities.
- Moreover, there is a need to inform and educate healthcare professionals regarding the use of traditional medicine. Some patients take traditional medicine and do not inform their healthcare provider. This is largely due to the stigma around the use of traditional medicine, thus resulting in drug–herb interactions.
- TB is an opportunistic infection, the risk of infection increasing in HIV-positive patients. There is, therefore, a need to conduct drug interactions, especially to ensure the safe use of traditional medicine in HIV-positive patients. This thus creates a gap in pharmacovigilance studies to develop criteria for each countries’ MRA.
- For most isolated compounds, there was no progress made from the study of extracts, phytochemical profiling to isolation, and in vitro studies, as well as little progress made in few in vivo studies, to identify compounds. Most studies end there; however, there is a need for basic research that will enable further higher-level studies to be performed, such as clinical studies using animal models, pharmacodynamic and pharmacokinetic studies, and quality assurance of traditional medicines in general.
- The number of deaths from TB remains high, despite all of the interventions such as the direct observed therapy (DOT) program, which involves healthcare workers, or other designated people, making sure that patients take their medicine correctly, thereby ensuring adherence and tolerability. According to the World Health Organization, the identification of TB cases increased after the Coronavirus Disease 2019 (COVID-19) pandemic due to the renewed attention toward infectious diseases other than COVID-19.
- TB deaths, however, remain high, especially in economically burdened countries. Governments may provide treatment; however, there are challenges around food insecurity and access. Without effective nutrition, the immune system is weakened and this increases the risk of active TB. Adherence to TB treatment thus proves to be difficult due to lack of food and the multiple drugs they have to take, resulting in resistance and, consequently, death.

## Figures and Tables

**Table 1:** Different drug regimens for drug-susceptible pulmonary TB for adults [7].

Regimen	Initial Phase		Continuation Phase		Doses
	Drugs	Dosing Frequency	Drugs	Dosing Frequency	
1	Isoniazid Rifampicin Pyrazinamide Ethambutol	7 d/w or 5 d/w (8 weeks)	Isoniazid Rifampicin	7 d/w or 5 d/w (18 weeks)	130–182 doses over 26 weeks
			Isoniazid Rifampicin	2 d/w (18 weeks)	76–92 doses over 26 weeks
			Isoniazid Rifapentine	** 1 d/w (18 weeks)	58–74 doses over 26 weeks
2	Isoniazid Rifampicin Pyrazinamide Ethambutol	7 d/w for 2 weeks then 2 d/w for 6 weeks or 5 d/w then 2 d/w for 6 weeks	Isoniazid Rifampicin	2 d/w (18 weeks)	58–62 doses over 26 weeks
			Isoniazid Rifapentine	** 1 d/w (18 weeks)	40–44 doses over 26 weeks
3	Isoniazid Rifampicin Pyrazinamide Ethambutol	3 times/week (18 weeks)	Isoniazid Rifampicin	54 doses 3 times/week (18 weeks)	78 doses over 26 weeks
4	Isoniazid Rifampicin Ethambutol	7 d/w for 8 weeks or 5 d/w for 8 weeks	Isoniazid Rifampicin	7 d/w for (31 weeks)	195–273 doses over 39 weeks
			Isoniazid Rifampicin	2 times/week (31 weeks)	102–118 doses over 39 weeks

Note: d/w—days per week. “Regimens given less than 3 times a week are not recommended for HIV-infected patients with CD4+ counts less than 100 cell/mm^3^”. ** Regimen for HIV-negative patients with negative sputum smears at the end of 2 months of therapy and without cavitation on an initial chest x-ray. For patients started on this regimen found to have positive culture from the 2-month specimen, treatment should be extended by an additional 3 months.

**Table 2:** Recommended dose in adults and children [7].

Dose mg/kg (Maximum Doses in mg)							
Drug	Group			Daily	Once Weekly	Twice Weekly	Thrice Weekly
Isoniazid	Adults			5 (300)	15 (900)	15 (900)	15 (900)
	Children			10–15 (300)		20–30 (900)	
Rifampicin	Adults			10 (600)		10 (600)	
	Children			10 (600)		10 (600)	
Rifabutin	Adults			5 (300)		5 (300)	
	Children			Unknown			
Rifapentine	Adults				10 (600) continuation phase		
	Children			No approval for use in group			
Pyrazinamide	Adults	Weight (kg)	40–55	18.2–25 (1000)		36.4–50 (2000)	27.3–37.5 (1500)
			56–75	20–26.8 (1500)		40–53.6 (3000)	33.3–44.6 (2500)
			76–90	22.2–26.3 (2000)		44.4–52.6 (4000)	33.3–39.5 (3000)
	Children			15–30 (2000)		50 (2000)	
Ethambutol	Adults	Weight (kg)	40–55	14.5–20 (800)		36.4–50 (2000)	21.8–30 (1200)
			56–74	16–21.4 (1200)		37.3–50 (2800)	26.7–35.7 (2000)
			76–90	17.8–21.1 (1600)		44.4–52.6 (4000)	26.7–31.6 (2400)
	Children			15–20 (1000)		50 (2500)	

**Table 4:** Phytoconstituents identified in *C. edulis*.

Compound Number	Structure	Compound Name	Phytochemical	Bioactivity	Antimycobacterium *M. tuberculosis*	References
**1**	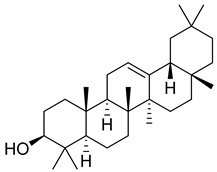	β-amyrin	Triterpene	Anti-inflammatory, antinociceptive, antioxidant, antipruritic, gastroprotective, hepatoprotective	*M. tuberculosis* H37Rv MIC > 200 mg/L	[25,26,27]
**2**	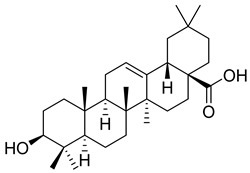	Oleanolic acid	Triterpenoid	Antioxidant, anti-tumor, anti-inflammatory, anti-diabetic, anti-microbial, hepatoprotective	*M. tuberculosis* H37Rv MIC 100 mg/L	[25,28]
**3**	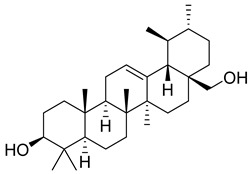	Uvaol	Triterpene	Antioxidant, anti-inflammatory, vasodilator	*M. tuberculosis* H37Rv MIC > 200 mg/L	[25,29,30]
**4**	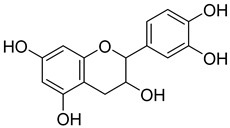	Epicatechin	Flavanol	Neuroprotective effects, blood-pressure-lowering effect	*M. tuberculosis* H37Rv MIC > 200 mg/L	[25,31,32]
**5**	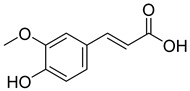	Ferulic acid	Phenolic	Anti-inflammatory, antibacterial, anticancer, anti-arrhythmic, antithrombotic		[33,34]
**6**	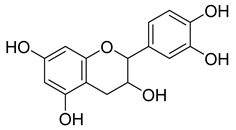	Catechin	Polyphenolic	Antioxidant, ultraviolet (UV) protection, antimicrobial, anti-allergy, anti-inflammatory, antiviral, anticancer	*M. tuberculosis* H37Rv MIC 200 mg/L	[25,35,36]
**7**	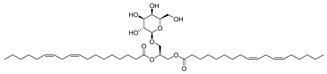	Monogalactosyldiacylglycerol (MGDG)	Galactolipid	Anti-inflammatory	*M. tuberculosis* H37Rv MIC > 200 mg/L	[25,37,38]
**8**	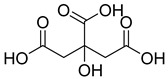	Citric acid	Phenolic	Antimicrobial	NR	[39,40]
**9**	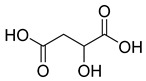	Malic acid	Phenolic	Antibacterial	NR	[40,41]
**10**	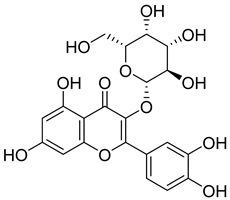	Hyperoside	Flavonoid	Antioxidant, analgesic, anticancer, neuroprotective, kidney protective	NR	[42,43]
**11**	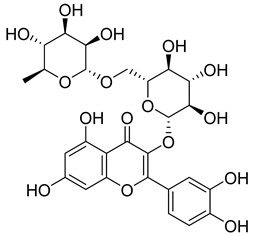	Rutin	Flavonoid	Anticancer, anti-inflammatory, neuroprotective, antiproliferative, antimetastatic, antioxidant, antimicrobial, antiallergy, antidiabetic	*M. tuberculosis* H37Rv MIC 25 µg/mL	[44,45,46,47]
**12**	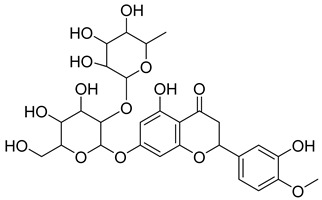	Neohesperidin	Flavonoids	Anti-inflammatory	NR	[48]
**13**	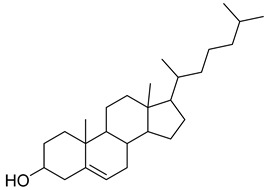	procyanidin B5	Polyphenol	Anti-inflammatory, anti-arthritic, anti-allergen	NR	[49]
**14**	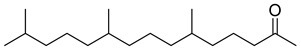	2-pentadecanone, 6,10,14-trimethyl	Sesquiterpene	Antibacterial, anti-nociceptive, anti-inflammatory	NR	[50]
**15**	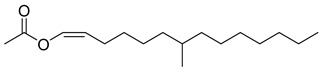	7-Methyl-Z-tetradecen-1-ol acetate	NR	NR	NR	
**16**	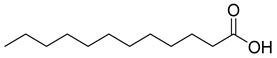	Dodecanoic acid	Fatty acid	Antibacterial	NR	[51]
**17**	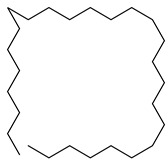	Heptacosane	Alkane		NR	[23]
**18**	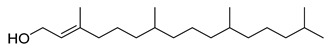	Phytol	Diterpene	Anxiolytic, cytotoxic, antioxidant, antinociceptive, antimicrobial, anti-inflammatory, immune modulating	NR	[52,53]
**19**	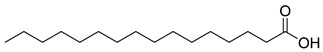	*n*-Hexadecanoic acid	Fatty acid	Antioxidant, hypocholesterolemic, nematicide, pesticide	NR	[53]
**20**	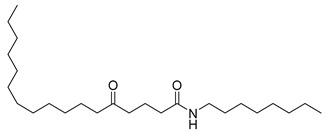	*n*-Octyl-5-oxoheptadecanamide	Amide	NR	NR	[23]
**21**	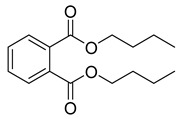	Dibutyl phthalate	NR	NR	NR	[23]
**22**	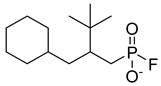	2-Tertbutyl cyclohexylpropylphosphonofluoridate	NR	NR	NR	[23]
**23**	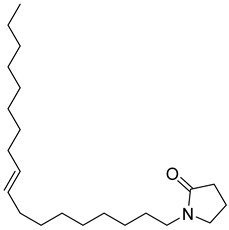	2-Pyrrolidinone, 1-(9-octadecenyl)	NR	NR	NR	[23]
**24**	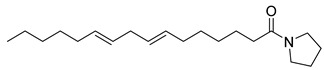	Pyrrolidine, 1-(1-oxo-7,10-hexadecadienyl	NR	NR	NR	[23]
**25**	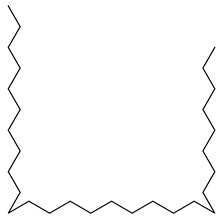	Nonacosane	Alkane	NR	NR	[23]
**26**	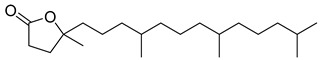	4,8,12,16-Tetramethylheptadecan-4-olide	Terpene	NR	NR	[54]
**27**	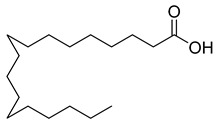	Octadecanoic acid	Fatty acid	Used to harden soaps, soften plastics, and make cosmetics, candles, and plastics	NR	[55]
**28**	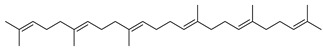	2,6,10,14,18,22-Tetracosahexaene, 2,6,10,15,19,23-hexamethyl	NR	NR	NR	[23]
**29**	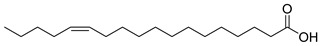	cis-13-Octadecenoic acid	Fatty acid	NR	NR	[23]
**30**	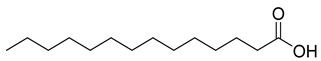	Tetradecanoic acid	Fatty acid	NR	NR	[54]
**31**	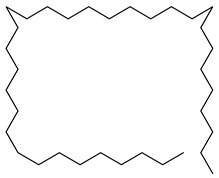	Tetratriacontane	Alkane	NR	NR	[23]
**32**	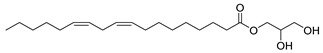	9,12-Octadecadienoic acid (Z,Z)-2 3-dihydroxypropyl ester	Ester	NR	NR	[23]
**33**	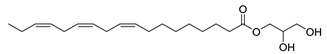	9,12,15-Octadecatrienoic acid, 2,3-dihydroxypropyl ester, (Z,Z,Z)	Ester	NR	NR	[23]
**34**	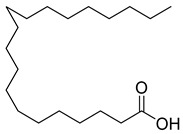	Eicosanoic acid	Fatty acid	NR	NR	[23]
**35**	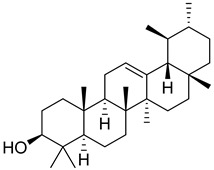	α-Amyrin	Triterpene	NR	NR	[26]
**36**	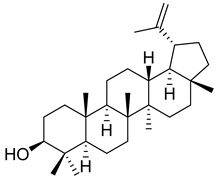	Lupeol	Triterpene	Antiprotozoal, antimicrobial, anti-inflammatory, antioxidant, antidiabetic, antitumor, chemopreventive, wound healing	*M. tuberculosis* H37Rv MIC > 20 µg/mL	[56,57,58]
**37**	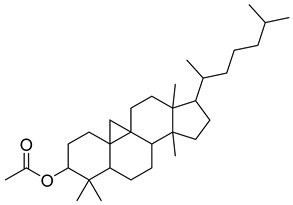	9,19-Cyclolanostan-24-en-3-ol, acetate, (3β)	NR	NR	NR	[23]
**38**	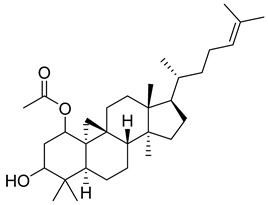	9,19-Cyclolanost-24-en-3-ol, acetate, (3β)	NR	NR	NR	[23]
**39**	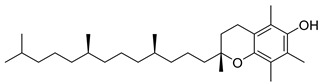	D-α-Tocopherol (Vitamin E)	Alpha tocopherol	Antioxidant, antidiabetic, UV irradiation protections	NR	[53,59]
**40**	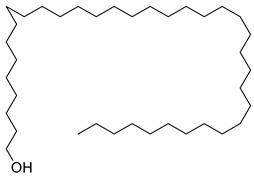	1-Heptatriacotanol	Alcohol [60]	Antioxidant, anti-inflammatory hypocholesterolemic, antimicrobial, anticancer [60]	NR	[23]
**41**	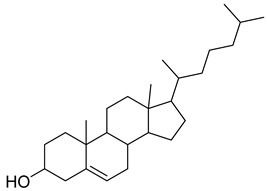	17-(1,5-Dimethylhexyl)-10,13-dimethyl- 2,3,4,7,8,9,10,11,12,13,14,15,16,17-tetradecahydro-1H-cyclopenta[a]phenanthren-3-ol	NR	NR	NR	[23]
**42**	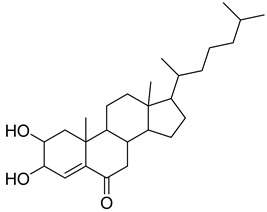	17-(1,5-Dimethylhexyl)-2,3-dihydroxy-10,13-dimethyl- 1,2,3,7,8,9,10,11,12,13,14,15,16,17-tetradecahydrocyclopenta[a]phenanthren-6-one	NR	NR	NR	[23]
**43**	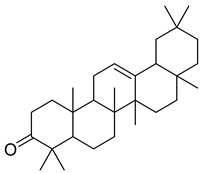	4,4,6a,6b,8a,11,11,14b-Octamethyl-1,4,4a,5,6,6a,6b,7,8,8a,9,10,11,12,12a,14,14a,14b-octadecahydro-2H-picen-3-one	NR	NR	NR	[23]

NR—Not reported at this time.

**Table 5:** *Drosera capensis* uses, biological effects, and phytochemistry.

Plant Part	Uses	Extraction		Biological Effect	Phytochemistry		References
	Traditional	Method	Type of Extract		Analysis/Profile	Bioactive Components	
Leaves	Fever, TB	Successive extraction with Ethanol	Ethanol extract	Ethanol leaf extract showed activity against *M. smegmatis* with MIC value of 3.125 mg/mL with isoniazid control MIC of 2 × 10^4^ mg/mL No activity against *M. tuberculosis*, control Ciprofloxacin MIC of 0.156 mg/mL and MBC of 0.312 mg/mL [65]	NR	Flavonoids	[17,65]
		Soxhlet (dried sample) Sonication (fresh sample) [67]	Methanolic extract	NR	NR	Plumbagin (**44**)	[67]

NR—Not reported at this time.

**Table 6:** Phytoconstituents identified in *Drosera capensis*.

Compound Number	Structure	Compound Name	Phytochemical	Bioactivity	Antimycobacterium *M. tuberculosis*	Mechanism of Action	References
**44**	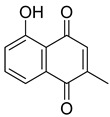	Plumbagin	Naphthoquinone	Antioxidant, anti-inflammatory, anticancer, antimicrobial, neuroprotective, antidiabetic, anti-atherosclerosis, analgesic	*M. tuberculosis* H37Rv MIC 21.3 µM MIC 4 µg/ml	Kills mycobacterial cells primarily by targeting ThyX, an enzyme required for their survival	[69,72,73,74]
**45**	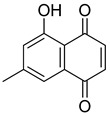	7-Methyljuglone	Naphthoquinone	Antifungal, antimicrobial, antitubercular, antiviral, anticancer	*M. tuberculosis* H37Rv MIC 0.50 µg/mL	NR	[75,76]
**46**	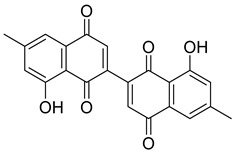	Mamegakinone	Naphthoquinone	NR	*M. tuberculosis* H37Rv MIC 100.00 µg/mL	NR	[77,78]
**47**	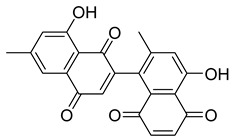	Neodiospyrin	Naphthoquinone	NR	*M. tuberculosis* H37Rv MIC 10.00 µg/mL	NR	[75,77]
**48**	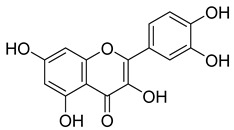	Quercetin	Flavonoid	Anti-inflammatory, antibacterial, antiviral, anticancer, neurodegenerative disorders, cardiovascular disease prevention, anti-allergy	*M. tuberculosis* H37Rv MIC 6.25 µg/mL [47]	Inhibits subunit B of DNA gyrase and isocitratelyase	[47,70]
**49**	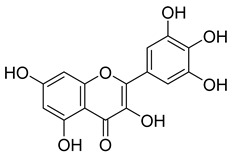	Myricetin	Flavonol	Antimicrobial, antioxidant, neurobiological activity, antidiabetic, anticancer, immunomodulatory, antihypertensive, cardioprotective, wound healing	*M. tuberculosis* H37Rv MIC 50.00 µg/mL	NR	[79]
**50**	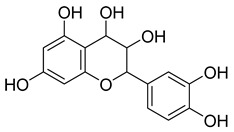	Leucocyanidin	Flavonoid	NR	NR	NR	[68]
**51**	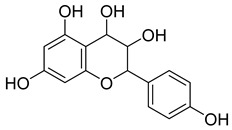	Leucopelargonidin	NR	NR	NR	NR	[68]
**52**	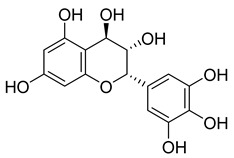	Leucodelphinidin	NR	NR	NR	NR	[68]
**53**	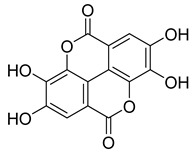	Ellagic acid	Polyphenol	Anti-mutagenic, antimicrobial, anticancer, HIV inhibition	NR	NR	[80]

NR—Not reported at this time.

**Table 7:** *Pelargonium reniforme* uses, pharmacological effects, and phytochemistry.

Plant Part	Uses	Extraction		Biological Effect	Phytochemistry		References
	Traditional	Method	Type of Extract		Analysis/Profile	Bioactive Components	
Tuber	Cough, TB	-	Ethanol, acetone, chloroform	Acetone, chloroform and ethanol root extracts showed activity against *M. tuberculosis* with MIC value of 10.3 mg/mL	NR	Scopoletin (**67**)	[17]
Roots	Cough, TB, diarrhea		Acetone, chloroform and ethanol Acetone and ethanol extract	Extracts inhibitory activity against drug-sensitive *M. tuberculosis* at 5 mg/mL Extracts active against fungal pathogens at 5 mg/mL	NR	NR	[84]
NR	NR	NR	NR	Antioxidant activity with IC_50_ ranging from 2.6 to 32.9 µM	NT	Gallic acid (**54**), methyl gallate (**55**), glucogallin (**56**), corilagin (**57**), vitexin (**58**), isovitexin (**59**), orientin (**60**), isoorientin (**61**), vitexin 2″-gallate (**62**), sovitexin 2″-gallate, orientin 2″-gallate (**66**), isoorientin 2″-gallate, quercetin (**63**), isoquercitrin (**64**), and rutin (**65**)	[87]

NR—Not reported at this time.

**Table 8:** Phytoconstituents identified in *Pelargonium reniforme*.

Compound Number	Structure	Compound Name	Phytochemical Class	Bioactivity	Antimycobacterium *M. tuberculosis*	Mechanism of Action	Reference
**54**	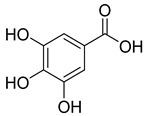	Gallic acid	Phenolic acid	Antioxidant, antimicrobial, anticancer, anti-inflammatory	*M. tuberculosis* H37Rv MIC_90_ 100 µM	NR	[79,88,89]
**55**	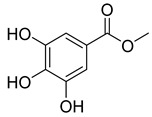	Methyl gallate	Phenolic	Antioxidant, anti-inflammatory, antimicrobial, diuretic	NR	NR	[90]
**56**	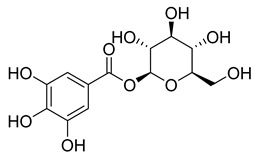	Glucogallin	-	-	NR	NR	[87]
**57**	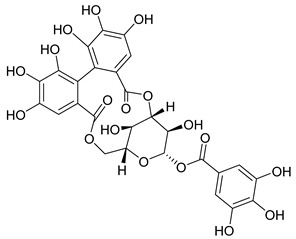	Corilagin	Polyphenol	Anticancer	NR	NR	[91]
**58**	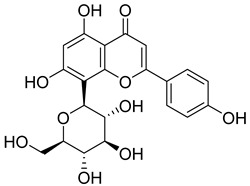	Vitexin	Mono-C-glycoflavone	Antidiabetic, antioxidant, anti-inflammatory, anticancer, antimicrobial, neuroprotective, cardioprotective	NR	NR	[92,93]
**59**	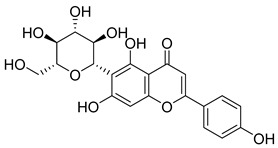	Isovitexin	Mono-C-glycoflavone	Antidiabetic	NR	NR	[92]
**60**	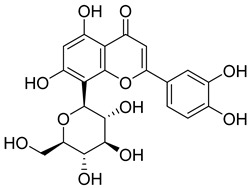	Orientin	Flavone	Anti-inflammatory, antioxidant, anticancer, anti-diabetes, analgesic	*M. tuberculosis* H37Rv MIC_50_ 23.4 ± 1.2 µg/mL (52.1 µM)	NR	[94,95]
**61**	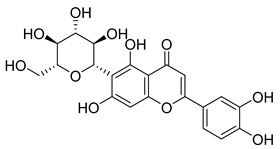	Isoorientin	Flavonoid	Anticancer	NR	NR	[96]
**62**	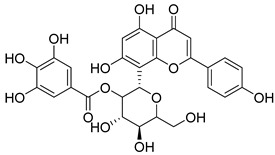	Vitexin 2″-O-gallate	NR	NR	NR	NR	[87]
**63**	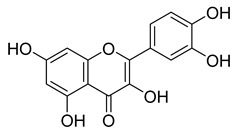	Quercetin	Flavonoid	Anti-inflammatory, antibacterial, antiviral, anticancer, neurodegenerative disorders, cardiovascular disease prevention, anti-allergy	*M. tuberculosis* H37Rv MIC 6.25 µg/ml	Inhibits subunit B of DNA gyrase, inhibits β-ketoacyl ACP synthase III involved in the synthesis of mycolic acid	[47,70]
**64**	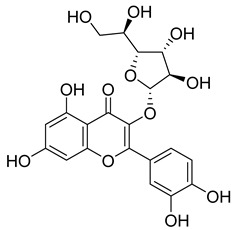	Isoquercitrin	Flavonoid	Anti-inflammatory, anti-allergy, anti-hyperlipidemic, antioxidant, antifungal	*M. tuberculosis* H37Rv MIC 0 µg/mL	NR	[97,98]
**65**	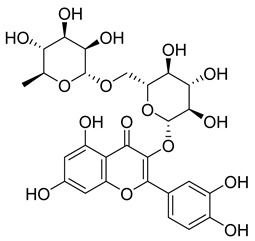	Rutin	Flavonoid	Anticancer, anti-inflammatory, neuroprotective, antiproliferative, antimetastatic, antioxidant, antimicrobial, antiallergy, antidiabetic	*M. tuberculosis* H37Rv MIC 25 µg/mL	NR	[44,45,46,47]
**66**	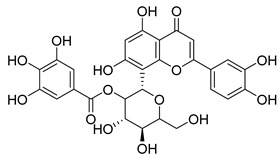	Orientin 2-O-gallate	Flavone	NR	NR	NR	[94]
**67**	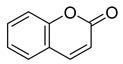	Scopoletin	Coumarin	NR	*M. tuberculosis* H37Rv MIC ≥ 100 µg/mL	NR	[98,99]

NR—Not reported at this time.

**Table 9:** *Tulbaghia violacea* uses, pharmacological effects, and phytochemistry.

Plant Part	Uses	Extraction		Biological Effect	Phytochemistry		References
	Traditional	Method	Type of Extract		Analysis/Profile	Bioactive Components	
Rhizome	Asthma, cold, fever, chest complaints, cough, influenza, sinusitis, TB, lung ulceration [17]	Maceration	Methanolic extract	NR	GC-MS	Beta-1,5-O-Dibenzoyl-ribofuranose (**81**), 4-Methoxybenzaldehyde (**82**), Oleyl alcoho, trifluoroacetate (**83**), Disulfide, bis(2-sulfhydrylethyl)- (**84**), Benzene, 1-methyl-4-(methylthio)- (**85**), 2,4-Dithiapentane (**73**), n-propyl 9,12-octadecadienoate (**87**), Methyl 5,13-Docosadienoate (**89**)	[17,108,109]
		Soxhelt extraction	Hexane extract	NR	IR, MS, NMR	Asymmetrically substituted novel dialkyl sulphone, deoxygenated analog of dialkyl sulphone, putative alkyl thiosulphinates	[107]
		Hydro-distillation	Essential oil	Antioxidant activity, cytotoxic effect, alternative source of anticancer, antibiotic, and antimicrobial agents [109]	GC-MS	Dimethy trisulfide (**86**) Dimethy disulfide, methyl (methylthio) meth 2,4-dithiapentane (Methylthio) acetic acid (**88**) (Methylthio) acetic acid, 2-(methylthiol) ethanol, propanitrile, 3-(methylthio)- 2,4dithiapntane,bis-(methlythio), disulfide	[109]
Bulbs	Enema for stomach problems	-	Aqueous extract	Antifungal activity against *Candida albicans* with an MIC value of 3.25 mg/mL	NR	NR	[13,105]
Leaves	Esophagus cancer	Maceration/homogenized Boiling	Acetone, Water	Ability to scavenge free radicals, IC_50_ for acetone extract at 207.33 µg/mL (68%) and IC_50_ for water extract at 168.88 µg/mL (73%) Anticancer effect, acetone extract indicated a dose-dependent inhibitory effect on human oral cancer cells, IC_50_ for acetone at 0.2 mg/mL and IC_50_ for aqueous extract at 1 mg/mL	NR	NR	[13,110]

NR—Not reported at this time.

**Table 10:** Phytochemical constituents identified in *T. violacea*.

Compound Number	Structure	Compound Name	Phytochemical/Class	References
**68**	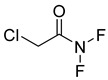	Chlorodifluoro acetamide	Acetic amide	[104]
**69**	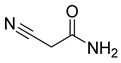	Acetamide, 2-cyano	Acetic amide	[104]
**70**	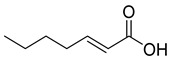	(E)-2-heptenoic acid	Fatty acid	[104]
**71**	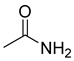	Acetamide	Acetic amide	[104]
**72**	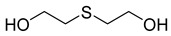	Thiodiglycol	Organosulfur compound	[104]
**73**	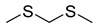	2,4-Dithiapentane	Organosulfur compound	[104]
**74**	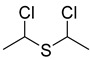	Chloromethyl sulfide	Sulfur compound	[104]
**75**	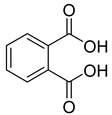	Phthalic acid	Dicarboxylic acid	[104]
**76**	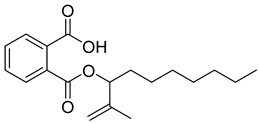	Phthalic acid, heptyl2-methylallyl ester	Dicarboxylic acid ester	[104]
**77**	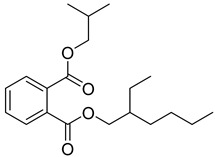	Phthalic acid 2-ethylhexyl isobutyl ester	Dicarboxylic acid ester	[104]
**78**	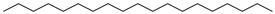	Nonadecane	Alkane	[104]
**79**	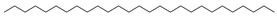	Heptacosane	Alkane	[104]
**80**	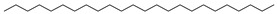	Tetracosane	Alkane	[104]
**81**	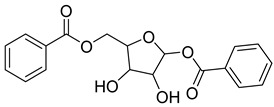	Beta-1,5-O-Dibenzoyl-ribofuranose	Pentose	[108]
**82**	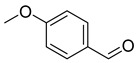	4-Methoxybenzaldehyde	Aldehyde	[108]
**83**	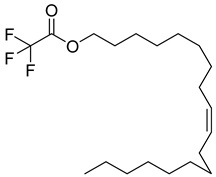	Oleyl alcohol, trifluoroacetate	Ester	[108]
**84**	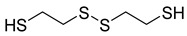	Disulfide, bis(2-sulfhydrylethyl)-	Organosulfur compound	[108]
**85**	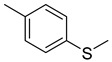	Benzene, 1-methyl-4-(methylthio)-	Sulfur compound	[108]
**86**	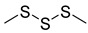	Dimethyl trisulfide	Organosulfur compound	[108]
**87**	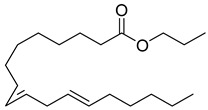	n-propyl 9,12-octadecadienoate	Linoleic acid	[108]
**88**	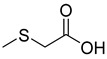	(Methylthio) acetic acid	Acetic acid	[108]
**89**	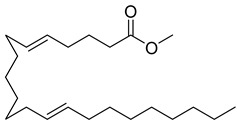	Methyl 5,13-Docosadienoate	Ester	[108]

## Data Availability

All data are included in the manuscript.

## Abbreviations

The following abbreviations are used in this manuscript:

COVID-19	Coronavirus Disease 2019
DOT	direct observed therapy
TB	Tuberculosis
HIV	Human Immunodeficiency Virus
MRAs	Medicine Regulatory Authorities
SAHPRA	South African Health Products Authority
MDR	multi-drug resistant
XRD	extensive-drug resistant
